# Aliens Among Us: Sensitivity of the Invasive Alien Fish Black Bullhead *Ameiurus melas* as a Bioindicator of Pollution and Its Safety for Human Consumption

**DOI:** 10.3390/toxics12120849

**Published:** 2024-11-25

**Authors:** Jovana Kostić, Jelena Đorđević Aleksić, Željka Višnjić-Jeftić, Dušan Nikolić, Zoran Marković, Margareta Kračun-Kolarević, Aleksandra Tasić, Milica Jaćimović

**Affiliations:** 1 Department of Biology and Inland Waters Protection, Institute for Multidisciplinary Research, University of Belgrade, 11030 Belgrade, Serbia; jelenadjo@imsi.rs (J.Đ.A.); zvisnjic@imsi.rs (Ž.V.-J.); dusan@imsi.rs (D.N.); mpucar@imsi.rs (M.J.); 2 Department of Growing and Reproduction of Domestic and Raised Animals, Institute of Animal Science, Faculty of Agriculture, University of Belgrade, 11080 Belgrade, Serbia; zoranmm@agrif.bg.ac.rs; 3 Department of Hydroecology and Water Protection, Institute for Biological Research “Siniša Stanković” National Institute of the Republic of Serbia, University of Belgrade, 11108 Belgrade, Serbia; margareta.kracun@ibiss.bg.ac.rs; 4 Scientific Institute of Veterinary Medicine of Serbia, 11107 Belgrade, Serbia

**Keywords:** pesticide pollution, invasive alien fish, comet assay, micronucleus assay, toxic elements, nature-based solutions

## Abstract

This study aims to evaluate the black bullhead *Ameiurus melas*, an invasive alien fish (IAF) in Serbia, as a bioindicator organism and assess the safety of natural and aquaculture specimens for human consumption. A set of biomarkers was analysed to assess the bioindicator potential at a site exposed to agricultural activities. The genotoxic response was determined by an alkaline comet assay and micronucleus assay in fish erythrocytes, and the metal pollution index (MPI) was calculated to assess the toxic element burden on fish. Water quality was evaluated using physicochemical parameters and faecal indicator bacteria, while sediment was analysed for the presence of pesticides. The concentration of metals and metalloids in fish muscle was monitored to assess the safety for human consumption, and the corresponding indices (MAC, THQ, HI) were calculated. All biomarker responses were linked by the integrated biomarker response (IBR). Water analyses indicated the absence of communal wastewater, while sediment analysis revealed the presence of paclobutrazol, bifenthrin, and cyfluthrin. The IBR showed that June and September had the highest stress indices, coinciding with peak pesticide use and precipitation. All indices confirmed the safety of black bullhead for human consumption. This study highlighted the uses of nature-based solutions to the problem of IAF.

## 1. Introduction

Covering less than 1% of Earth’s surface, freshwater ecosystems are biodiversity hotspots, hosting one-third of vertebrate species and 10% of all species [1], providing, at the same time, ecosystem services to billions of people [2]. Despite efforts and initiatives, the loss of biodiversity dramatically continues to occur, representing one of the most severe human-caused global environmental problems [3,4,5]. After habitat destruction, invasive alien fish (IAF) are a second cause of the reduced biodiversity in aquatic environments and the potential extinction of local fish populations [6,7]. In the face of climate change, declining biodiversity weakens the resilience of aquatic ecosystems, demanding immediate attention [8].

The black bullhead *Ameiurus melas* [9], a species native to North America, was introduced to Western Europe in the late 19th century, mainly for aquaculture and sport fishing. During this time, there was a growing interest in stocking European waters with exotic fish species to improve recreational fishing opportunities and provide anglers with a greater variety of fish [10]. This species was recorded in Serbia for the first time in 2005 [11], and today, it has become ubiquitous in the environment. Thanks to several life-history and ecological characteristics, such as high fecundity, parental care of offspring, prolonged reproductive period, high flexibility in life-history traits, voracious and versatile feeding habits, habitat and water-quality flexibility, and tolerance to pollution, black bullhead are a very successful invader [12,13,14,15,16,17,18,19]. In terms of IAF management, their use as bioindicator organisms [20,21], as well as their safety for consumption and aquaculture potential, should be examined [22,23]. By monitoring the response of different biomarkers in IAF at the locality of interest, information could be obtained about their condition and the possible mechanisms that allow them to be superior to native populations. The alkaline comet assay is a widely used and sensitive method for detecting genotoxic potential, indicating the level of initial DNA damage in individual eucaryotic cells [24]. If the specimens are chronically exposed to stressful conditions, their repair mechanisms can weaken, and permanent chromosomal damage may occur [25]. The micronucleus assay detects micronuclei (MNs) in the cell’s cytoplasm. These structures represent either whole or fragmented chromosomes smaller than a third of the primary cell nucleus [26]. This assay can uncover additional nuclear abnormalities (NAs), including binuclei and lobed, blebbed, notched, and irregular nuclei [27]. Pollution with metals and metalloids poses a risk to the environment and human health. The degree of contamination and burden on fish can be expressed by the metal pollution index (MPI) [28]. With regard to nutrition and human health, of particular importance is the analysis of certain elements in fish muscle for which maximum allowable concentrations (MACs) are calculated [29,30]. In addition, the total hazard quotient (THQ) and hazard index (HI) can be determined to assess the potential health risks of fish consumption to humans [31].

Within the frame of the Rufford Foundation project “Mass removal of the black bullhead (*Ameiurus melas*)—Possibilities for self-sustaining commercial farming in Serbia” (ID: 31053-2), this study was conducted at Markovac Lake to examine the potential of the black bullhead, an IAF in Serbia, as a bioindicator organism and evaluate its safety for human consumption. A set of biomarkers was analysed to achieve this. The genotoxic response was assessed by an alkaline comet assay and a micronucleus assay in blood cells, and the MPI was calculated to assess the burden of toxic elements on fish. To reveal the safety of incorporating natural and cultivated black bullhead in the human diet, the concentration of metals and metalloids in fish muscle was monitored, and appropriate indices were calculated (MAC, THQ, HI). The Integrated Biomarker Response v.2 (IBRv.2) method was applied to obtain a comprehensive overview of environmental stress in comparison to control (aquaculture) fish. To assess water quality and identify primary pollution sources, complementary analyses included basic physicochemical parameters, microbiological indicators of faecal pollution, and an examination of pesticides in the lake sediment.

## 2. Materials and Methods

### 2.1. Sampling Site and Procedure

**Figure 1:**
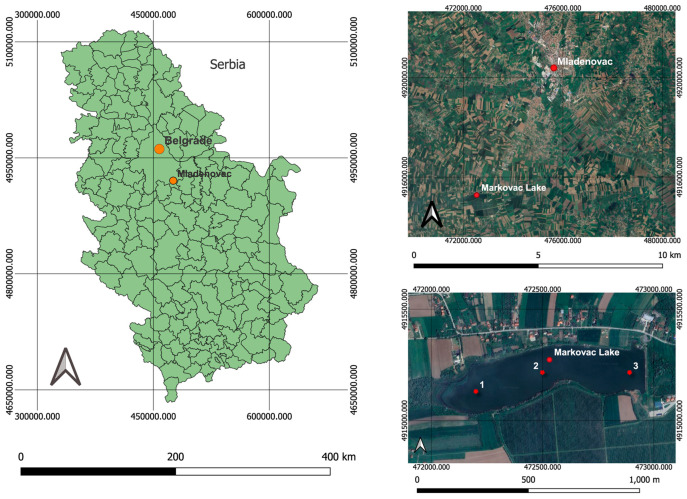
Sampling site Markovac Lake with sediment sampling points (1, 2 and 3). Map created using the Free and Open Source QGIS Version 3.24.0.

Markovac Lake (44°23′23.3″ N 20°39′14.2″ E) (Figure 1) is located at the foot of Kosmaj Mountain. It is one of the oldest and richest artificial reservoirs in the territory of Belgrade (municipality Mladenovac), the capital of Serbia. It was created in the mid-1960s to meet the irrigation needs of the nearby agricultural combine and apple orchard. The lake is 1000 m long and 500 m wide, with an average depth of 7 m. On the slopes of this lake, one of the largest apple orchards in that part of Serbia (50 ha) is located. This is also a very attractive location for tourists and recreational fishermen. A sampling of water and fish was carried out in the months of June (15/06/21), July (06/07/21), August (24/08/21), and September (14/09/21). These months were selected as they align with the typical application period for pesticides throughout the year [32].

### 2.2. Analyses of Water

Every month, water samples were collected in sterile glass bottles to analyse the basic physicochemical parameters, including temperature, pH, oxygen concentration and saturation, conductivity, and total dissolved solids (TDS), as well as nitrite (NO_2_^−^), nitrate (NO_3_^−^), and ammonium (NH_4_^+^) concentrations. Additionally, faecal indicator bacteria, specifically total coliforms and *E. coli*, were monitored. Physical parameters were measured with a multiparameter sonde YSI 6600 V2 (Xylem Analytics, Sydney, Australia). Chemical parameters were determined by using an AQUANAL™-plus test set (Sigma Aldrich, Seelze, Germany). The absorbance level was measured with a WinLab Data Line Photometer (Winlab, Clausthal-Zellerfeld, Germany), using the following methods: “Nitrite +” (0.01–2 mg/L NO_2_^−^), “Nitrate low +” (0.1–30 mg/L NO_3_^−^), and “Ammonium +” (0.05–3.0 mg/L NH_4_^+^). Water was classified according to the “Regulation on parameters of ecological and chemical status of surface waters and parameters of chemical and quantitative status of groundwater” [33], and reference values for accumulations formed on type 3 and type 4 water bodies were used to determine water-quality class. Based on the results, the water was classified into specific quality categories, which were described as excellent (I), good (II), moderate (III), poor (IV), and bad (V). Total coliforms and *E. coli* were simultaneously detected and quantified by the ISO 9308-2:2012 standard [34] Colilert-18 test (IDEXX, Westbrook, ME, USA). Water quality according to the faecal indicator bacteria was determined based on the classification of Kavka et al. and Kirschner et al. [35,36].

### 2.3. Fish Sampling

Once per month, 10 specimens of black bullhead (wild-caught fish) were sampled using double fyke nets (length 82 cm, diameter 50 cm, and hole size 8 mm) that had been in the water for 24 h. Before dissection, specimens were anaesthetised with clove oil (50 µL/L) and euthanised by a quick blow to the head, applied above the brain. Total length L (cm) and weight W (g) were measured, and Fulton’s condition factor (K) was calculated according to the formula K = 100 × (W/L^3^) [37]. For the comet and micronucleus assay, blood was taken directly from the heart with a heparinised 2 mL syringe and 21G needle. A part of the muscle was sampled for the analysis of micro- and macroelements using ICP-OES. As a control, 10 black bullhead specimens (aquaculture fish) reared in a recirculating aquaculture system (RAS) in the Centre for Fisheries and Applied Hydrobiology (CEFAH) of the University of Belgrade, Faculty of Agriculture, were processed (19/10/21) in the same way as the samples from Markovac Lake. Experimental breeding of these individuals lasted from June to October 2021.

### 2.4. Alkaline Comet Assay

An alkaline comet assay was performed based on our previous work [38] with slight modifications. In brief, on site, a drop of blood was placed in a cold 1× PBS solution and transported to the laboratory within one hour in a dark and cool box (4 °C). Samples were additionally diluted 20× in cold 1× PBS. The cell suspension was mixed with 1% LMP agarose (low melting point), placed on the 1% NMP (normal melting point) layer on the glass slide previously coated with 1% NMP, and covered with a coverslip. After the gel solidified (5 min, 4 °C), the coverslips were removed, and the slides were placed in an ice-cold lysis solution (2.5 M NaCl, 100 mM EDTA, 10 mM Tris, 1% Triton X-100, 10% DMSO, pH 10, at 4 °C for 16–18 h). After lysis, denaturation (20 min, 4 °C) and electrophoresis (0.75 V cm^−1^, 300 mA, 20 min, 4 °C) were performed in an electrophoresis chamber using an alkaline solution (300 mM NaOH, 1 mM EDTA, pH 13). The slides were then neutralised (0.4 M Tris, pH 7.5, 15 min, 4 °C) and fixed in cold methanol (15 min, 4 °C). For the positive control, cells embedded in agarose were exposed to 20 µM H_2_O_2_ for 15 min [38], washed with 1× PBS, and further processed as described above.

Samples were stained with 1:500-diluted GreenSafe Premium (Nzytech, Lisbon, Portugal), and comet-like structures were observed under 400× magnification using a DM4 B fluorescence microscope (Leica, Wetzlar, Germany) equipped with a DFC7000 T camera (Leica, Wetzlar, Germany). Images were saved in “.bmp” format and manually scored in CometScore 2.0 software (TriTek Corporation, Sumerduck, VA, USA). The DNA damage was expressed by using the Tail DNA (%) parameter, which represents one of the most common descriptors of DNA damage level [39].

### 2.5. Micronucleus Assay

For the micronucleus assay, a drop of blood was spread on the pre-cleaned slide, air dried for one hour, and fixed in cold methanol for 15 min. GreenSafe Premium (Nzytech, Lisbon, Portugal), diluted 1:500, was used for staining. Cells were observed under 400× magnification using a DM4 B fluorescence microscope (Leica, Wetzlar, Germany) equipped with a DFC7000 T camera (Leica, Wetzlar, Germany). For each sample, 2000 cells were randomly selected, and the number of micronuclei and other nuclear aberrations was expressed in parts per thousand (‰).

### 2.6. Analysis of Micro- and Macroelements in Muscle

Samples of muscle tissue were rinsed with distilled water and stored at −18 °C until processed for element analysis. Approximately 0.5 g of dry weight (dw) muscle tissue sample was processed in a microwave digester (ETHOS EASY Advanced Microwave Digestion System 230 V/50 Hz, Milestone, Bergamo, Italy) with 6 mL of 65% HNO_3_ (Suprapur^®^, Merck, Darmstadt, Germany) and 4 mL of H_2_O_2_ (Suprapur^®^, Merck, Darmstadt, Germany). After cooling to room temperature, digested samples were diluted with distilled water to a total volume of 25 mL. The concentrations of 23 elements (Al, As, Ba, B, Cd, Cr, Co, Cu, Pb, Li, Se, Ag, Sb, Mo, Pt, Rh, Sn, Ti, Sr, Fe, Zn, Mn, and Ni) were assessed using ICP-OES (Inductively Coupled Plasma—Optical Emission AVIO 200, PerkinElmer, Shelton, CT, USA). The concentrations of all elements are expressed as mg kg^−1^ dry weight (dw). The element values are expressed in mg kg^−1^ wet weight for calculating the indices MPI, THQ, and HI.

Blank samples resolved the potential presence of the analysed elements in the chemicals used for digestion. Table S1 presents the ICP-OES method’s detection wavelength (λ) and lower detection threshold (mg/L).

The target hazard quotient (THQ) was calculated using the following formula:

THQ = ((Efr × ED × FIR × C)/(Bwa × AT × RfD)) × 10^−3^ (1)

where Efr is the exposure frequency (365 days/year), ED is the exposure duration (70 years), FIR is the daily quantity of human consumption of freshwater fish (fish intake rate), which is 4.2 g per person per day in Serbia [40], C is the element concentration in fish muscle (μg/g wet weight), RfD is the oral reference dose (Hg = 0.0005, Cd = 0.001, Pb = 0.004, Cu = 0.04, Zn = 0.3, Cr = 1.5, Mn = 0.14, Al = 0.0004, As = 0.0003, Fe = 0.04, Co = 0.0003, Ni = 0.02 μg/g ww), WAB is the average body weight of an adult (70 kg), and TA is the average exposure time (365 days/year × ED) [41,42,43].

The hazard index (HI), or the total THQ, was calculated as the sum of individual THQ values for each element:

HI = THQ1 + THQ2 + THQn(2)

The metal pollution index (MPI) was calculated to evaluate the total bioaccumulation degree in the muscle of fish specimens, using the following formula [44]:

MPI = (M1 × M2 × … × Mn)^1/n^ (3)

where Mn is the concentration of metal n (in muscle tissue). The MPI employed potentially toxic trace elements and heavy metals, including Al, Ba, Cd, Cr, Co, Cu, Pb, Sr, Fe, Zn, Mn, and Ni.

The concentration of elements expressed in the wet weight (ww) was compared to the maximum allowable concentration (MAC) prescribed by the EU regulations, national regulations of RS, and FAO recommendations. According to the European Commission Regulation, the maximum acceptable concentrations (MACs) for Cd, Pb, and Hg in fish meat are 0.05 µg/g, 0.3 µg/g, and 0.5 µg/g ww, respectively [45]. The national regulations of the Republic of Serbia prescribe 1.0, 0.1, 2.0, 1.0, 30.0, 30.0, and 100.0 µg/g ww as the MACs for Pb, Cd, As, Hg, Cu, Fe, and Zn in fish meat, respectively [46]. The limits recommended by the Food and Agriculture Organization for both Cu and Zn are 30 µg/g ww [47].

### 2.7. Sediment Analysis

Sediment sampling for the analysis of pesticides was subsequently conducted in April 2023. The sediment samples were collected at three representative sampling points designated as 1 (44°23′20.14″ N, 20°39′3.46″ E), 2 (44°23′22.95″ N, 20°39′16.96″ E), and 3 (44°23′23.00″ N, 20°39′34.69″ E) (Figure 1). The pesticides in sediment were examined to optimise and validate a modified extraction method based on QuEChERS [48]. This method is used to quantitatively determine multiple pesticide residues in sediment, whereby pesticides currently used most frequently in agriculture were primarily investigated. The validated method accurately quantified 90 pesticides and metabolites by gas chromatography with mass detection (GC/MS).

#### 2.7.1. Sample Extraction

The reagents used in this study were of chromatographic grade and were obtained from Merck (Darmstadt, Germany). The solid pesticide standards and the internal standard (triphenyl phosphate) were obtained from Dr. Ehrenstorfer™ (Augsburg, Germany). Dissolution of the individual undiluted pesticides in acetone or acetonitrile yielded stock solutions of 1 mg/mL, which were used to prepare a mix stock solution of 10 μg/mL in acetonitrile, which was further diluted to a 1 μg/mL working standard mixture. Table S2 lists the 90 pesticides and metabolites analysed in this study. It also contains information on pesticide action and retention time as well as the qualifier and quantifier ions for each particular residue. Sediment samples were oven-dried at 60 °C and sieved at 250 μm before residue testing. For extraction, 10 g of sample was weighed into a 50 mL PTFE centrifuge tube, and 10 mL of acetonitrile was added. Then, 100 µL of internal standard (10 µg/mL) was added. The QuEChERS salts used for extraction were 4 g of MgSO_4_ and 1 g of NaCl for the first phase of extraction, while the following QuEChERS extraction salts obtained from Agilent (Santa Clara, CA, USA) were used for the second phase of purification: 150 mg of C18, 150 mg of primary–secondary amine (PSA), and 900 mg of MgSO_4_. After adding the MgSO_4_ salt and NaCl in the first phase, the mixture was shaken for 1 min and then vortexed by a Mini Vortex Stirrer LBX Instruments, V03 series (Labbox Labware, Barcelona, Spain). Afterwards, the reaction mixture was centrifuged for 5 min (at 4000 rpm). An aliquot of 8 mL was transferred from the supernatant to a new, clean 15 mL centrifuge tube containing the sorbents of 150 mg PSA, 150 mg C18, and 900 mg anhydrous MgSO_4_ for a procedure termed dispersive solid-phase extraction (d-SPE) cleanup. After re-centrifugation, the top layer of extracts was finally collected in the vials and dried under a gentle stream of nitrogen gas. After removal of the interferences through a nylon filter, the residues after evaporation were dissolved in 1 mL of ethyl acetate: hexane (1:1) and analysed using a GC-MS instrument.

#### 2.7.2. GC-MS Analysis

The analysis of pesticides was conducted using a gas chromatograph (GC) system Clarus 680 (PerkinElmer, Shelton, CT, USA) equipped with a Clarus SQ8T mass spectrometer (PerkinElmer, Shelton, CT, USA). The injector operated at 250 °C. A capillary column (Elite-5MS, 30 m, 0.25 mm I.D., 0.25 μm film thicknesses, 5% phenyl 95% dimethylpolysiloxane) was used to separate the target analytes. Helium (ultrahigh purity) was used as the carrier gas at a constant pressure of 20 psi. The initial oven temperature of 70 °C was held for 3 min, increased to 150 °C at 23 °C/min, then to 200 °C at 3 °C/min, further increased to 280 °C at 7 °C/min, and then maintained at the final temperature of 280 °C for 9 min. The solvent delay time was 5.0 min. The injection volume was 1.0 μL in the spitless mode. The mass spectrometer (MS) inlet line and the ion source temperatures were set at 280 °C and 250 °C, respectively. The MS ionization energy was 70 eV. Pesticides in the extracts from the sediment samples were analysed in the selected ion monitoring mode (SIM) using one target and three qualifier ions for each analyte (Table S2). The limit of quantification for all analytes was 5 µg kg^−1^ dry weight. All analyses were conducted in triplicate, and the results are expressed as the mean ± SD.

#### 2.7.3. Method Validation and Accuracy Assurance

The data quality for pesticide residues was ensured by examining solvent blanks, procedural blanks, internal standards, detection limits, qualification limits, and certified reference material. To eliminate the influence of interference, all reagents used in the analysis were subjected to identical extraction techniques and checked on the GC/MS system before analysis. Certified reference material and blank samples were prepared according to the same procedure and recorded in the same run as the analysed sediment samples. The calibration parameters are presented in Supplementary Table S3. The limit of detection (LOD) was determined using the signal-to-noise ratio (0.0005 mg kg^−1^), whereas the limit of quantification (LOQ) was determined using recovery and precision data (set at 0.005 mg kg^−1^ for each pesticide residue). The matrix effect was studied considering the calibration curves in the solvent (ethyl acetate: hexane (1:1)) and the matrix. The calculation based on the equation %Mes = ((peak area (matrix standard)/peak area (solvent standard)) − 1) × 100 [48] showed that the matrix effect was less than 20%. For quantification, calibration was used in both the matrix and the solvent, given that the instrument software allows it. The recovery parameters for a low calibration spike of 5 µg kg^−1^ ranged from 74.5 to 115.2%, and for a higher spike of 50 µg kg^−1^, the range was 75.9–119.6% (Table S3). This confirmed a satisfactory condition for the successful optimisation and validation of the method according to the EU SANTE/12682/2019 guidelines [49], as it falls within the 70–120% range. All concentrations of detected pesticides in sediment samples were represented on a dry weight basis (μg/kg dw).

### 2.8. Integrated Biomarker Response (IBR) Analysis

Integrated Biomarker Response v.2 (IBRv.2) analysis was performed in Microsoft Excel based on the method of Sanchez et al. [50] with minor modifications. All sampling months for Markovac Lake and reference site Radmilovac were ranked using radial diagrams constructed based on the selected parameters (condition index—CI, micronucleus frequency—MN, TI% comet assay—CA, metal pollution index—MPI, and hazard index—HI). Considering all possible arrangements of the parameters on the radial diagrams, the mean value and standard deviation for each IBR area were also calculated.

### 2.9. Statistical Analyses

For statistical analyses, IBM SPSS Statistics Version 25 (North Castle, NY, USA) was used. Whether the samples were normally distributed was determined using a Shapiro–Wilk test. Statistical differences in the condition index were examined with a one-way ANOVA. Significant differences between samples were determined using a Kruskal–Wallis H test for the comet assay data. Differences between the sampling months and control for nuclear aberrations and IBR were determined using a paired-samples *t*-test (normal distribution) and a Wilcoxon signed-rank test (not-normal distribution). For element concentrations and MPI where data did not follow a normal distribution (Al, Ba, Cr, Co, Cu, Li, Pt, Ti, Sr, Fe, Zn, and Mn), significance was tested using a Mann–Whitney U test, while for normally distributed data (Cd, Pb, Se, and Ni), a paired-sample *t*-test was used. For all tests, the significance level was set to 0.05.

## 3. Results

### 3.1. Analyses of Water

Table 1 gives the results on the basic physicochemical parameters and the microbiological indicators of faecal pollution in the water of Markovac Lake during four sampling months.

The highest water temperature, TDS concentration, and water conductivity and the lowest O_2_ concentration were measured in July. Based on the pH values, the water was classified in the III-IV category, except in September, when the water fell into the IV-V category. Oxygen concentrations pointed to the II-III water-quality class, except July, when it belonged to the IV-V category. According to the NH_4_^+^ concentrations, the water belonged to category II-III, except in September, when it was classified as the III-IV category. Based on the NO_3_^−^ levels, water was classified in the II-III category. The concentration of EC was below 100 MPN/100 mL during all of the months, pointing to the I class of water quality. According to TC, water quality was in the I category in July, the II category during June and August, and the III class in September.

### 3.2. Fulton’s Condition Factor

Table 2 shows the results of fish average total length L (cm) and weight W (g) for the control and four sampling months, as well as Fulton’s condition factor (K). There were no statistically significant differences in fish condition between the examined groups.

### 3.3. Alkaline Comet Assay

**Figure 2:**
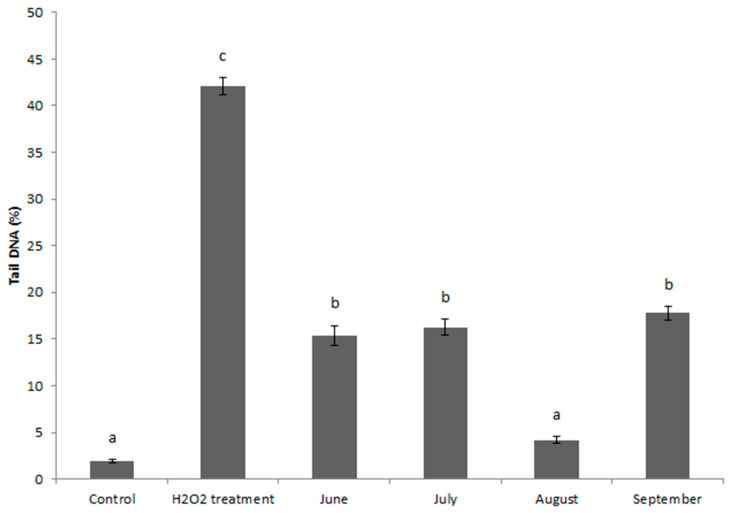
The level of DNA damage during sampling months, at the control and after H_2_O_2_ treatment (positive control). ^a,b,c^—statistical significance is indicated by different letters among groups (*p* < 0.05); groups with the same letters are not significantly different.

The degree of DNA damage determined by the alkaline comet assay is shown in Figure 2. A statistical significance was found between all samples and the H_2_O_2_ treatment of control site blood cells. In all months except August, the DNA damage level was significantly higher than the control. There were no significant differences in DNA damage level between June, July, and September.

### 3.4. Micronucleus Assay

The results of the micronucleus analysis are presented in Table 3. Micronuclei were the most represented type of nuclear aberrations. The highest number of nuclear aberrations, including micronuclei, was observed in June, while the control showed the lowest number of micronuclei and most other nuclear abnormalities. A statistically significant higher number of micronuclei compared to the control were observed in June, August, and September.

**Figure 3:**
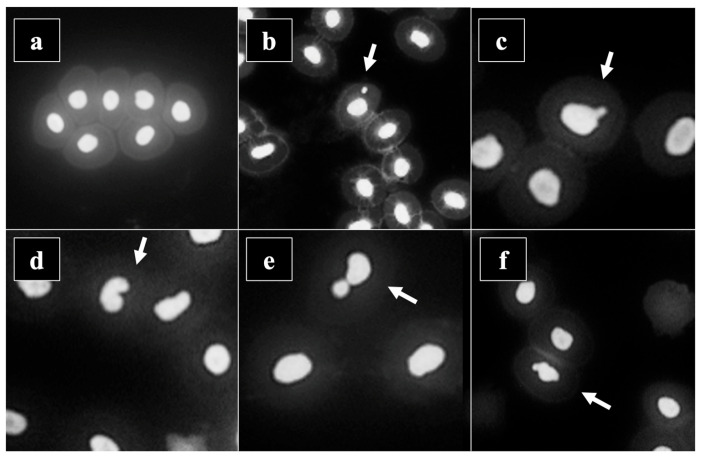
Nuclear aberrations analysed by the micronucleus assay ((**a**)—normal cells, (**b**)—micronucleus, (**c**)—bud-shaped nuclei, (**d**)—notched nuclei, (**e**)—binucleus, (**f**)—irregularly shaped nuclei).

Figure 3 shows the types of nuclear aberrations that were observed during this study.

### 3.5. Analysis of Micro- and Macroelements in Muscle

The concentrations of As, B, Ag, Sb, Mo, Rh, and Sn were below the detection limit. In June, we observed significantly higher values of Ba, Cu, Li, Sr, and Zn compared to the control. During July, Cd, Mn, and Ni had significantly higher values than the control. In August, Mn and Ni had significantly higher concentrations in comparison to the control, while in September, significant differences were observed for Sr and Ni (Table 4).

The HI value did not exceed 1 in either control (aquaculture) or wild-caught fish, so the use of black bullhead meat in the human diet poses no risk. The values for Al were the highest and had a multiplicative influence on the value of HI, while the other elements (Cd, Cr, Co, Cu, Pb, Fe, Zn, Mn, and Ni) had much smaller effects. The lowest THQ values were obtained for Cr (Control: 0.0000038; June: 0.0000047; July: 0.0000030; August: 0.0000026; September: 0.0000032), probably due to the high reference value (RFD). The HI and MPI had the highest values during June but without a statistical significance between months and the control (Table 5).

Black bullhead meat from both aquaculture and native populations is safe for human consumption as none of the detected elements (Pb, Cd, Cu, Fe, and Zn) exceed the recommended MAC values. The concentrations of As and Hg were below the detection threshold (Table S4).

### 3.6. Sediment Analysis

Among the 90 tested residues, only the pesticide paclobutrazol was detected at sampling point 1 (11.56 ± 0.69 µg kg^−1^). At sampling point 2, pesticides bifenthrin (5.75 ± 0.39 µg kg^−1^) and cyfluthrin (41.38 ± 4.48 µg kg^−1^) were discovered, while none of the 90 tested residues were detected at sampling point 3.

### 3.7. IBR Analysis

**Figure 4:**
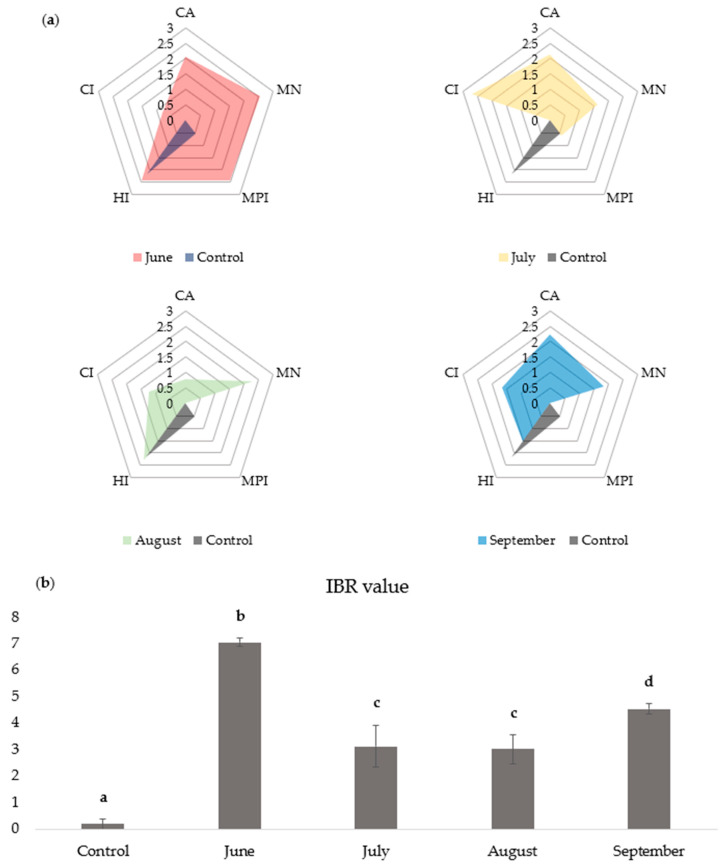
IBR test results: (**a**) IBR diagram for each sampling month compared to the control for all examined biomarkers: condition index (CI), metal pollution index (MPI), hazard index (HI), comet assay (CA), micronucleus assay (MN); (**b**) IBR diagram values for the sampling months for all parameters (mean ± SD); ^a–d^—different letters denote significant differences (*p* < 0.05).

The results of the IBR analysis for all the inspected biomarkers across all sampling months at Markovac Lake, compared to the control, are presented in Figure 4. It was observed that the impact of different biomarkers on the expansion of the IBR diagram area varied depending on the month. A statistically significant increase in the IBR diagram area was observed in all months compared to the control. The highest stress index with an IBR value of 7.06 ± 0.16 was observed in June. This was attributed to the highest levels of genotoxicity alongside the highest MPI and HI. In contrast, the control group showed the lowest IBR value of 0.19 ± 0.20, with all biomarkers displaying a weaker response compared to Lake Markovac, except for the HI.

## 4. Discussion

Having in mind nature-based solutions (NBSs) and circular economy, the problem of IAF could be solved through their use as bioindicators, as a food source in aquaculture, or for animal feed production [51,52,53,54]. To find successful strategies for managing IAF, this study was carried out to investigate the potential of the black bullhead, an invasive alien fish in Serbia, as a bioindicator organism and assess the safety of both natural and aquaculture specimens for human consumption.

The Fulton’s condition factor is often used in ecological monitoring to indicate the general condition of fish. This approach is fast, simple, and inexpensive, but it lacks sensitivity [55]. The values of K were similar to those reported for European introduced populations (K = 1.37) of black bullhead [18]. The absence of statistical significance between the control and different months in this study confirmed its low sensitivity, as observed in other studies [56,57,58].

Genotoxicity biomarkers were chosen due to their high sensitivity and ability to bridge biomarkers at lower and higher levels of biological organisation [57,59]. Several studies have used brown bullhead, a closely related species, as a reliable bioindicator for genotoxicity [60,61,62,63,64]. To our knowledge, this is the first study to apply a comet and MN assay to test the genotoxic response in black bullhead.

In our field tests, we chose a site impacted by extensive agricultural activities exposed to pesticide pollution through surface runoff. Analysis of *E. coli* ruled out the presence of municipal wastewater at the site. However, we confirmed the presence of paclobutrazol, bifenthrin, and cyfluthrin in the lake sediment in April 2023, highlighting pesticides from the apple orchard on the lake shore as the primary source of pollution. It is important to note that pesticides can cause significant harm, especially when applied in mixtures, leading to synergistic effects on various endpoints such as DNA damage, chromosomal aberrations, toxicity, oxidative stress, apoptosis, and effects on detoxifying enzymes and hormones [65,66]. Paclobutrazol is commonly used as a plant growth regulator during spring and early summer and can also be applied later in the summer [67]. However, there have been no studies on its genotoxic effects. Nevertheless, research has indicated its potential to disrupt behaviour, organ development, and the biomarkers of antioxidant defence, oxidative response, SOD, and neurotransmitter levels in the brain of zebrafish [68,69]. Bifenthrin, a pyrethroid insecticide and acaricide, primarily exerts neurotoxic effects [70,71]. It is often applied in spring and fall and is considered persistent in the environment, with high toxicity and bioaccumulation potential in aquatic organisms [72,73,74]. Ullah et al. [75] classified bifenthrin as highly toxic for grass carp (*Ctenopharyngodon idella*), disrupting multiple biomarkers, including DNA damage. Cyfluthrin, another pyrethroid, has been proven to be toxic to the crustaceans *Daphnia magna* and *Ceriodaphnia dubia*, even at low concentrations of 0.07 µg/L [76], and zebrafish embryos [77]. The study of Marinowic et al. [78] demonstrated the genotoxic effects of β-cyfluthrin in the erythrocytes of the fish *Bryconamericus iheringii*. Our results point to consistent levels of DNA damage throughout June, July, and September, which coincides with the months of pesticide application. The intriguing twist in August, with a statistically significant decrease in DNA damage, could be attributed to the reduced precipitation during that month, which caused a reduced input of pesticides into the lake water [32,79]. These findings emphasise the intricate relationship between environmental factors and pesticide impact. Unlike the comet assay, the micronucleus test detects permanent chromosomal damage, classified as aneugenic or clastogenic depending on whether the whole or part of the chromosome is lagging [80,81]. Both assays effectively evaluate pollution-induced stress and cytotoxicity in aquatic ecosystems [82,83,84,85]. Regarding MN and nuclear abnormalities, our study showed the highest level of these aberrations during June. Many studies have shown the potential of pyrethroid insecticides to induce micronucleus and nuclear aberrations in fish blood [83,86,87]. It should be borne in mind that the presence of micronuclei is influenced not only by the repair capacity of the organism [88] but also by cell kinetics and cell replacement, i.e., the process of erythropoiesis [89,90].

Determining the concentration of heavy metals in fish muscle is the first step in assessing the level of contamination in the aquatic ecosystem and evaluating its impact on human health [91]. Fish consumption is proven to lead to several health benefits, due to its antioxidant, anti-inflammatory, wound healing, neuroprotection, cardioprotection, and hepatoprotection properties. Fish proteins, such as immunoglobins, act as defence agents against viral and bacterial infections [92]. However, due to the presence of toxic metals, consuming fish meat can be harmful; therefore, it is necessary to examine the health impact of such a diet [42,93]. This is especially important considering that there is limited data available in the literature on wild-caught black bullhead specimens of an allochthonous species. According to the obtained results regarding MAC and HI, the muscle of black bullhead is considered safe for human consumption. However, the MPI values in the muscle of this species from wild and aquaculture (0.49–0.69) populations are moderately high and several times higher compared to other studies on native fish from different artificial reservoirs in Serbia [42,94]. This indicates the need for more detailed research into the risks associated with using this IAF for human consumption. Similar to our findings, many studies have shown higher concentrations of metals and other toxic elements in the tissues of wild-fish specimens compared to their aquaculture counterparts [95,96,97]. Needless to say, specimens reared in aquaculture are raised in a safe and controlled environment, protected from both natural and anthropogenic sources of stress. The elevated Mn levels in tissues may result from the increased concentration and bioavailability of this element in the lake water, likely due to a pH decrease during the summer months. Similarly, the rise in Cu concentration in muscle tissue could be attributed to ecological stress in the lake’s aquatic system, as Cu levels in blood and tissue increase rapidly in response to elevated concentrations in the water [98]. Given that the processes determining the accumulation of Sr in fish depend on ecological and biological factors such as the type of geological substrate, water chemistry, and trophic level, size, and age of fish [99], it is challenging to explain the cause of the difference in Sr concentration in the muscle of black bullhead between the wild and control samples. Several studies have also shown that different formulations of pesticides and insecticides may also contain metals in their composition [100,101,102]. For example, a high concentration of Ni is present in the commercial preparation Pyrinex [103], which also contains bifenthrin, the insecticide detected in this study. In this way, agricultural activity double burdens aquatic ecosystems with both organic and inorganic pollutants.

Integrated biomarker response is an analysis that combines all monitored biomarkers into a “general stress index” [104]. When the response of several biomarkers is examined at different localities, IBR allows a comprehensive overview of the specimens’ state, enabling clear distinction between sites [105]. In this study, IBR analysis revealed that June, followed by September, had the highest “general stress index”. This aligns with the months of high pesticide application and high precipitation conditions [32]. Further, IBR validated Radmilovac as an appropriate and reliable control site.

## 5. Conclusions

Regarding the use of black bullhead as a bioindicator, this study provided valuable data on its sensitivity and highlighted the impact of environmental conditions on the variability of biomarker response. It would be desirable to direct future research towards comparing the response of this species with that of native ones to assess its potential to replace them in biomonitoring studies. In addition, such studies would shed light on the possible resistance mechanisms of IAF compared to native populations.

Regarding incorporating this species into human nutrition and discussing its presence in the food supply, our findings indicate that both wild and cultivated specimens are safe for consumption. Further management steps could be focused on educating the aquaculture sector about the possibilities of breeding this species by utilising natural populations. Also, wild individuals of this species could be recommended for human consumption or the production of pet food after testing the nutritional value of their meat.

Both approaches represent a nature-based solution to the problem of IAF, creating a win–win solution.

## Figures and Tables

**Table 1:** Physicochemical parameters and microbiological indicators of water quality at Markovac Lake.

	June	July	August	September
T (°C)	21.7	25.8	23.5	19.7
pH	8.17	7.91	8.42	8.93
O_2_ (mg/L)	6.84	2.57	5.40	6.77
O_2_ (%)	77.5	32.3	63.0	74.4
Conductivity (μS/cm)	357	409	306	288
TDS (mg/L)	0.247	0.261	0.205	0.208
NO_2_^−^ (mg/L)	0.01	0.01	0.52	0.01
NO_3_^−^ (mg/L)	1.26	0.10	Br *	0.10
NH_4_^+^ (mg/L)	0.11	0.09	0.05	0.18
TC MPN/100 mL	763	381.5	2442	>12,098
EC MPN/100 mL	20	10	43	10

* br—below range.

**Table 2:** Total length and body weight of fish with Fulton’s condition factor.

	Control	June	July	August	September
L	25.2 ± 1.1	13.6 ± 1.5	13.5 ± 1.5	14.5 ± 1.6	16.0 ± 1.0
W	226.9 ± 45.3	34.7 ± 11.2	30.2 ± 8.7	41.2 ± 11.8	53.5 ± 10.8
K	1.4 ± 0.1	1.4 ± 0.3	1.2 ± 0.1	1.3 ± 0.1	1.3 ± 0.1

L—total length (cm), W—weight (g), K—Fulton’s condition factor. Values are presented as the mean ± SD.

**Table 3:** Frequency of micronuclei and nuclear abnormalities.

	Control	June	July	August	September
MN *	0.15 ± 0.34 ^a^	1.30 ± 0.89 ^b^	0.60 ± 0.61 ^ab^	1.08 ± 0.87 ^b^	0.70 ± 0.42 ^b^
BUD **	0.55 ± 0.44 ^ab^	0.85 ± 0.71 ^b^	0.33 ± 0.24 ^a^	0.75 ± 0.54 ^ab^	0.90 ± 0.61 ^b^
NOTCHED **	0.90 ± 0.61 ^a^	0.10 ± 0.21 ^b^	0.25 ± 0.63 ^ab^	0.20 ± 0.48 ^b^	0.23 ± 0.25 ^b^
BI **	0 ± 0 ^a^	0.15 ± 0.34 ^ab^	0.20 ± 0.26 ^b^	0.43 ± 0.44 ^b^	0.55 ± 0.72 ^b^
IRR **	0.05 ± 0.16	0.40 ± 0.61	0.20 ± 0.35	0.10 ± 0.21	0.25 ± 0.49

MN—micronucleus, BUD—bud-shaped nuclei, NOTCHED—notched nuclei, BI—binucleus, IRR—irregularly shaped nuclei presented as the mean ± SD in parts per thousand (‰). ^a,b^—different letters indicate statistically significant differences between the control and different months (*p* < 0.05); * paired-sample *t*-test; ** Wilcoxon signed-rank test.

**Table 4:** The concentration of elements in the muscle of the black bullhead specimens from the control and in different months (Lake Markovac).

	Control	June	July	August	September
Al	12.61 ± 18.32	34.05 ± 18.53	8.93 ± 12.41	15.50 ± 29.27	12.81 ± 29.12
Ba	2.18 ± 1.14	5.15 ± 1.08 *	2.97 ± 2.34	2.18 ± 0.99	3.30 ± 1.74
Cd	0.01 ± 0.02	0.06 ± 0.02	0.08 ± 0.02 *	0.04 ± 0.02	0.05 ± 0.02
Cr	0.42 ± 0.21	0.59 ± 0.28	0.37 ± 0.07	0.34 ± 0.12	0.39 ± 0.11
Co	0.17 ± 0.06	0.19 ± 0.09	0.11 ± 0.06	0.11 ± 0.07	0.09 ± 0.07 *
Cu	1.82 ± 0.37	4.79 ± 1.50 *	0.40 ± 0.34	2.19 ± 0.35	1.87 ± 0.40
Pb	0.60 ± 0.19	0.40 ± 0.12 *	0.41 ± 0.17 *	0.70 ± 0.23	0.58 ± 0.14
Li	0.04 ± 0.03	0.34 ± 0.08 *	0.07 ± 0.03	0.04 ± 0.03	0.60 ± 0.05
Se	0.71 ± 0.48	0.42 ± 0.31	0.73 ± 0.25	0.53 ± 0.35	0.86 ± 0.32
Pt	0.27 ± 0.13	0.25 ± 0.15	0.24 ± 0.16	0.25 ± 0.05	0.22 ± 0.11
Ti	0.31 ± 0.43	0.79 ± 0.55	0.28 ± 0.32	0.31 ± 0.66	0.16 ± 0.25
Sr	2.93 ± 2.69	11.77 ± 5.53 *	5.23 ± 3.76	3.75 ± 3.54	6.67 ± 6.25 *
Fe	33.03 ± 22.24	48.63 ± 30.84	33.22 ± 17.93	34.32 ± 24.92	30.45 ± 17.65
Zn	124.45 ± 28.82	204.98 ± 33.45 *	124.00 ± 32.24	109.04 ± 24.54	123.22 ± 40.58
Mn	3.54 ± 2.43	5.53 ± 5.93	11.42 ± 2.08 *	7.83 ± 4.97 *	6.28 ± 1.86
Ni	4.97 ± 2.39	8.93 ± 7.03	13.97 ± 2.21 *	10.79 ± 4.71 *	8.82 ± 1.85 *

Values are expressed in mg kg^−1^ dry weight (mean ± SD). * Statistical significance in comparison to the control (*p* < 0.05).

**Table 5:** Target hazard quotient (THQ), hazard index (HI), and metal pollution index (MPI) in the muscle tissue of black bullhead.

	Control	June	July	August	September
Al THQ	0.433	0.408	0.280	0.451	0.417
Cd THQ	0.014	0.012	0.009	0.005	0.006
Co THQ	0.077	0.074	0.046	0.043	0.036
Cu THQ	0.006	0.014	0.006	0.006	0.006
Pb THQ	0.002	0.001	0.001	0.002	0.002
Fe THQ	0.011	0.015	0.010	0.010	0.009
Zn THQ	0.006	0.008	0.005	0.004	0.005
Mn THQ	-	-	0.001	0.001	0.001
Ni THQ	0.034	0.054	0.085	0.061	0.055
HI	0.57	0.59	0.44	0.58	0.53
MPI	0.53	0.69	0.53	0.49	0.49

## Data Availability

The raw data supporting the conclusions of this article will be made available by the authors on request.

## Supplementary Materials

The following supporting information can be downloaded at: https://www.mdpi.com/article/10.3390/toxics12120849/s1, Table S1: Lower detection threshold (mg/L) and detection wavelength (λ) of the ICP-OES; Table S2: List of pesticide residues—information related to pesticide definition, chemical classification, and pesticide action, retention time, and quantifier and qualifier ions; Table S3: Validation parameters—calibration curves, correlation coefficients, linearity (range 5–50 µg/kg), recovery for two spiked levels, and relative standard deviations (RSDs); Table S4: Concentrations of elements in the muscle tissue of the black bullhead are expressed in mg kg^−1^ wet weight.
