# Supplementary material for: Aliens Among Us: Sensitivity of the Invasive Alien Fish Black Bullhead Ameiurus melas as a Bioindicator of Pollution and Its Safety for Human Consumption
Source: Toxics. 2024 Nov 25;12(12):849. doi: 10.3390/toxics12120849 (PMC11679168; doi:10.3390/toxics12120849)
Supplement: Supplementary file 1 [file toxics-12-00849-s001.zip › toxics-3299893-supplementary.pdf]

Table S1: Lower detection threshold (mg/L) and detection wavelength (λ) of the ICP-OES

	**Lower detection threshold (mg/L)**	**Detection wavelength *(λ)***
**Al**	0.01	396.153
**As**	0.02	193.696
**Ba**	0.01	233.527
**B**	0.02	249.677
**Cd**	0.01	228.802
**Cr**	0.005	267.716
**Co**	0.005	228.616
**Cu**	0.005	327.393
**Pb**	0.01	220.353
**Li**	0.002	670.784
**Se**	0.02	196.026
**Ag**	0.01	328.068
**Sb**	0.01	206.836
**Mo**	0.005	202.031
**Pt**	0.01	265.945
**Rh**	0.01	343.489
**Sn**	0.01	189.927
**Ti**	0.005	334.940
**Sr**	0.001	407.771
**Fe**	0.001	238.204
**Zn**	0.005	206.200
**Mn**	0.001	257.610
**Ni**	0.005	231.604

Table S2: List of pesticide residues – information related to pesticide definition, chemical classification and pesticides action, retention time, quantify and qualifier ions

**Analite**	**Chemical Class**	**Pesticide action***	**Retention time, min**	**Quantify trace**	**Qualifier ion,** 1 > 2 > 3	**Serial number**
**Dichlorvos**	Organophosphate	IN	5.45	109	187 >79 >185	1
**Acephate**	Organophosphate	IN	7.45	136	94 >95 >42	2
**Propoxur**	Carbamate	IN	9.15	110	152 >57 >111	3
**Omethoate**	Organophosphate	IN, AC	10.05	121	156 >110 >109	4
**Ethoprophos**	Organophosphate	IN, NE	10.39	158	97 >126 >139	5
**Diphenylamine**	Aromatic amine	FU, PG	10.48	137	169 >168 >170	6

**Chlorpropham**	Carbamate	PG, HB	11.20	148	127 >213>153	7
**Dioxabenzofos**	Organophosphate	IN	11.26	158	216 >183>201	8
**Alpha Lindan**	Organochlorine	IN	11.74	181	183 > 219>217	9
**Dimethoate**	Organophosphate	IN	12.34	194	93 > 125 >143	10
**Carbofuran**	Carbamate	IN, NE, AC	12.59	204	149 >131 >123	11
**Beta Lindan**	Organochlorine	IN	13.09	219	181 >183 >217	12
**Lindan**	Organochlorine	IN	13.14	181	183 >219 >111	13
**Diazinon**	Organophosphate	IN	13.78	179	137 >152 >199	14
**Delta HCH**	Organochlorine	IN	14.65	181	219 >183 >217	15
**Phosphamidon I**	Organophosphate	IN, AC	14.70	254	127 >264>72	16
**Pyrimethanil**	Anilinopyrimidine	FU	14.75	250	198 >199>200	17
**Phosphamidon II**	Organophosphate	IN, AC	16.2	327	127 >264>72	18
**Methyl Parathion**	Organophosphate	IN	16.28	263	109 >125 >79	19
**Spiroxamine I**	Morpholine	FU	16.33	100	101 >126 >198	20
**Heptachlor**	Organochlorine	IN	16.49	100	270 >272 >274	21
**Chlorpyriphos methyl**	Organophosphate	IN, AC	16.58	338	286 >288 >125	22
**Vinclozolin**	Dicarboximide	FU	16.63	340	212 >285>198	23
**Carbaryl**	Carbamate	IN, PG	16.66	144	115 >116 >145	24
**Metalaxyl**	Phenylamide	FU	16.77	206	45 >160 >249	25
**Isoproturon**	Phenylurea	HB	17.09	358	146 >72 >206	26
**Aldrin**	Organochlorine	IN	18.45	100	101 >126	27

					>198	
**Malathion**	Organophosphate	IN	18.26	173	127 >125 >93	28
**Spiroxamine II**	Morpholine	FU	18.23	263	66 >265 >261	29
**Pirimiphos-methyl**	Organophosphate	IN	18.31	233	290 >276 >305	30
**Chlorpyrifos**	Organophosphate	IN	18.46	197	199 >314 >97	31
**Heptachlor epoxide (trans, isomer A)**	Organochlorine	IN	20.70	351	353 >355 >357	32
**Pendimethali n**	Dinitroaniline	HB	20.99	252	253 >281 >162	33
**Captan**	Dicarboximide	FU	21.10	79	80 > 151 > 77	34
**Fipronil**	Aminopyrazole carbonitrile	IN	21.13	367	369 >213 >351	35
**Cyprodinil**	Anilinopyrimidine	FU	21.28	224	225 >210 >226	36
**Penconazole**	Triazole	FU	21.48	248	159 >161 >250	37
**Folpet**	Phthalimide	FU	21.59	104	76 >260 >147	38
**trans Chlordane**	Organochlorine	IN	21.79	373	375 >377 >371	39
**Procymidone**	Dicarboximide	FU	22.03	96	283 >285 >67	40
**Fluopyram**	Pyridylethylamide	FU, NE	22.14	173	145 >195 >223	41
**Prallethrin cis**	Pyrethroid	IN	22.17	123	105 >81 >134	42
**Prallethrin trans**	Pyrethroid	IN	22.24	123	81 >105 >77	43
**Paclobutrazol**	Triazole	PG	22.40	236	125 >238 >167	44
**Alpha Endosulfan**	Organochlorine	IN	22.43	241	195 >239 >238	45
**cis Chlordane**	Organochlorine	IN	22.47	373	375 >377 >371	46

**Flutriafol**	Triazole	FU	23.17	123	219 >164 >83	47
**Imazalil**	Imidazole	FU	23.40	41	215 >173 >217	48
**Hexaconazole**	Triazole	FU	23.53	83	214 >216 >82	49
**Fludioxonil**	Phenylpyrrole	FU	23.85	248	127 >154 >182	50
**Dieldrin**	Organochlorine	IN	23.72	79	263 >277 >279	51
**p, p' - DDE**	Organochlorine metabolite	IN	23.77	246	318 >316 >248	52
**Kresoxim methyl**	Strobilurin	FU	24.36	116	206 >131 >132	53
**Myclobutanil**	Triazole	FU	24.52	179	150 >181 >82	54
**Chlorfenapyr**	Halogenated pyrrole	IN	24.68	59	247 >249 >408	55
**Endrin**	Organochlorine	IN	24.60	263	317 >265 >245	56
**Buprofezin**	Thiadiazine	IN, AC	24.95	105	104 >106 >172	57
**Beta Endosulfan**	Organochlorine	IN	25.09	195	237 >207 >241	58
**Bupirimate**	Aminopyrimidine	FU	25.20	273	208 >316 >166	59
**Cyproconazole**	Triazole	FU	25.44	222	139 >224 >125	60
**p, p' - DDD**	Organochlorine metabolite	IN	25.49	235	237 >165 >236	61
**Oxadixyl**	Phenylamide	FU	26.21	105	163 >132 >77	62
**Endosulfan sulfate**	Organochlorine metabolite	IN	26.53	272	274 >229 >237	63
**Propiconazole I**	Triazole	FU	26.60	69	173 >259 >175	64
**Trifloxystrobin**	Strobilurin	FU	26.73	116	131 >59 >172	65
**Propiconazole II**	Triazole	FU	26.79	173	69 >259 >175	66
**p, p' - DDT**	Organochlorine	IN	26.77	235	237 >165	67

	metabolite				>236	
**Quinoxifen**	Quinoline	FU	26.85	237	272 >309 >307	68
**Tebuconazole**	Triazole	FU	27.28	125	250 >70 >83	69
**Spiromesifen**	Spirocyclic tetronic acid	IN	27.83	272	99 > 273 > 254	70
**Endrin ketone**	Organochlorine metabolite	IN	28.00	317	67 >315 >319	71
**Phosmet**	Organophosphate	IN	28.24	160	161 >77 >93	72
**Bifenthrin**	Pyrethroid	IN	28.43	181	165 >166 >182	73
**Fenoxycarb**	Carbamate	IN	28.56	255	186 >77 >185	74
**Metoxychlor**	Organochlorine	IN	28.60	113	152 >227 >228	75
**Etoxazole**	Diphenyloxazolin e	AC	28.64	141	204 >300 >359	76
**Permethrin I**	Pyrethroid	IN	31.04	183	163 >165 >184	77
**Pyridaben**	Pyridazinone	IN, AC	31.24	147	117 >148 >132	78
**Permethrin II**	Pyrethroid	IN	31.23	183	163 >165 >184	79
**Cyflutrin I**	Pyrethroid	IN	31.84	163	206 >165 >226	80
**Cyfluthrin II**	Pyrethroid	IN	31.99	163	206 >165 >226	81
**Cyfluthrin III**	Pyrethroid	IN	32.06	163	206 >165 >226	82
**Cyfluthrin IV**	Pyrethroid	IN	32.13	163	206 >165 >226	83
**Cypermethrin I**	Pyrethroid	IN	32.21	181	163 >165 >209	84
**Cypermethrin II**	Pyrethroid	IN	32.37	181	163 >165 >209	85
**Cypermethrin III**	Pyrethroid	IN	32.44	181	163 >165 >209	86
**Cypermethrin IV**	Pyrethroid	IN	32.51	181	163 >165 >209	87

**Boscalid**	Carboxamide aka anilide	FU	32.40	140	342 >344 >142	88
**Ethofenprox**	Pyrethroid	IN	32.75	163	164 >135 >107	89
**Azoxystrobin**	Strobilurin	FU	35.68	344	388 >345 >372	90

*AC – acaricide, IN – insecticide, NE – nematocide, FU –fungicide, PG – plant growth regulator, HB – herbicide

Table S3: Validation parameters – calibration curves, correlation coefficients, linearity (range 5 – 50 µg/kg), recovery for two spiked levels and and relative standard deviations (RSDr)

**Analite**	**Calibration curve**	**R^2**	**Spike 0.005 mg/kg[b]**		**Spike 0.050 mg/kg[b]**	
			**Rec (%)**	**RSDr (%)**	**Rec (%)**	**RSDr (%)**
**Dichlorvos**	y=4.474x -26.955	0.994591	85.2	5.94	89.2	6.12
**Acephate**	y=0.826x – 38.023	0.991543	89.2	1.94	93.7	3.22
**Propoxur**	y=7.484x – 157.22	0.998957	95.3	2.81	94.2	2.15
**Omethoate**	y=2.153x – 10.326	0.999482	102.9	5.89	103.2	4.58
**Ethoprophos**	y=1.822x – 50.872	0.991542	115.2	6.93	110.5	5.21
**Diphenylamine**	y=4.901x – 32.962	0.998422	114.2	11.94	116.8	7.49
**Chlorpropham**	y=1.486x – 9.846	0.991894	76.9	9.61	78.2	8.91
**Dioxabenzofos**	y=0.894x – 11.248	0.999877	77.4	1.13	81.5	2.61
**Alpha Lindan**	y=1.658x – 10.348	0.998972	95.2	3.24	99.7	3.44
**Dimethoate**	y=2.791x – 88.974	0.994717	96.8	3.01	96.2	3.94
**Carbofuran**	y=1.726x – 49.667	0.999944	97.5	1.77	95.4	2.14
**Beta Lindan**	y=1.239x – 10.425	0.997118	96.6	1.13	97.4	1.05
**Lindan**	y=1.355x – 6.084	0.997911	74.5	3.30	75.9	3.29
**Diazinon**	y=1.175x – 19.302	0.999655	109.2	1.78	109.1	2.94
**Delta HCH**	y=1.223x –	0.996887	107.6	1.50	115.2	1.97

	18.199					
**Phosphamidon I**	y=0.409x + 8.111	0.997948	112.3	6.74	119.6	3.64
**Pyrimethanil**	y=3.519x + 26.515	0.994892	74.8	10.7	94.2	7.79
**Phosphamidon II**	y=0.795x + 11.949	0.999489	75.8	6.71	78.1	6.15
**Methyl Parathion**	y=1.566x – 50.917	0.992631	85.2	5.45	85.1	6.93
**Spiroxamine I**	y=5.264x – 133.13	0.998944	82.9	2.53	83.9	6.11
**Heptachlor**	y=0.719x – 59.083	0.999948	88.5	2.98	94.1	3.01
**Chlorpyriphos methyl**	y=0.884x + 9.994	0.999773	88.9	9.19	99.0	2.76
**Vinclozolin**	y=0.445x – 9.789	0.999119	99.8	2.48	99.1	1.08
**Carbaryl**	y=1.881x – 210.91	0.999817	97.5	2.73	94.5	7.28
**Metalaxyl**	y=1.497x – 13.587	0.999499	94.5	3.04	93.2	2.45
**Isoproturon**	y=1.984x – 10.998	0.994795	94.2	1.52	95.8	1.38
**Aldrin**	y=0.507x – 3.657	0.991914	99.9	3.64	102.1	4.11
**Malathion**	y=2.136x – 87.645	0.999006	102.9	2.54	101.8	3.61
**Spiroxamine II**	y=8.345x – 552.90	0.998007	99.5	3.17	100.7	9.15
**Pirimiphos-methyl**	y=1.563x – 3.648	0.997981	107.4	3.91	108.1	10.52
**Chlorpyrifos**	y=1.531x – 93.506	0.998887	106.2	4.81	106.9	1.94
**Heptachlor epoxide (trans, isomer A)**	y=0.239x – 3.633	0.992524	93.7	6.72	95.1	14.35
**Pendimethalin**	y=0.556x – 45.370	0.998781	93.1	11.95	94.9	5.74
**Captan**	y=0.789x – 3.796	0.999789	105.2	10.94	110.5	8.52
**Fipronil**	y=0.441x – 11.649	0.998847	100.9	9.18	101.2	1.48
**Cyprodinil**	y=3.154x – 4.526	0.998788	98.7	8.94	103.2	1.92
**Penconazole**	y=1.547x –	0.997711	95.4	3.46	104.9	5.16

	55.849					
**Folpet**	y=0.903x – 78.799	0.995877	95.1	1.28	95.2	4.25
**trans Chlordane**	y=0.462x – 2.332	0.998275	103.4	2.84	103.7	3.23
**Procymidone**	y=2.470x – 52.523	0.995548	102.5	3.96	103.1	3.11
**Fluopyram**	y=3.156x – 17.526	0.994857	96.2	13.41	94.1	2.88
**Prallethrin cis**	y=0.821x – 18.406	0.993233	98.2	12.95	98.6	9.21
**Prallethrin trans**	y=1.038x – 42.422	0.991258	97.8	3.43	111.2	6.43
**Paclobutrazol**	y=0.957x – 30.544	0.993218	93.7	3.34	100.5	6.51
**Alpha Endosulfan**	y=0.288x – 5.093	0.995445	85.2	2.77	92.4	1.94
**cis Chlordane**	y=0.441x – 7.151	0.996777	85.9	1.64	87.1	1.94
**Flutriafol**	y=2.689x – 11.748	0.994879	88.9	10.60	89.5	6.47
**Imazalil**	y=0.534x + 0.882	0.993567	92.4	4.61	95.1	5.15
**Hexaconazole**	y=2.867x + 0.448	0.997888	94.1	14.12	94.6	2.31
**Fludioxonil**	y=1.092x – 22.616	0.996892	94.7	2.47	98.8	6.25
**Dieldrin**	y=2.838x – 27.454	0.999409	99.7	2.02	105.4	4.75
**p, p' - DDE**	y=2.067x – 9.143	0.999913	96.8	2.15	100.1	3.08
**Kresoxim methyl**	y=2.834x – 58.783	0.998837	97.5	5.79	100.9	1.99
**Myclobutanil**	y=2.677x – 23.511	0.996974	97.6	5.55	97.9	10.9
**Chlorfenapyr**	y=0.345x – 7.290	0.999715	98.4	4.36	99.3	1.05
**Endrin**	y=0.318x – 8.653	0.996120	106.4	2.94	106.1	1.90
**Buprofezin**	y=3.652x – 14.641	0.997945	103.5	1.08	100.0	2.05
**Beta Endosulfan**	y=0.308x – 5.381	0.998889	100.1	2.91	108.2	3.92
**Bupirimate**	y=1.941x – 11.052	0.998118	96.1	3.78	97.5	3.99
**Cyproconazole**	y=2.264x – 24.749	0.997748	93.3	3.17	95.8	5.21

**p, p' - DDD**	y=3.490x – 37.424	0.994687	93.9	3.08	94.8	4.17
**Oxadixyl**	y=3.155x – 21.899	0.993949	110.2	4.67	97.9	10.94
**Endosulfan sulfate**	y=0.282x – 6.980	0.998699	99.9	1.90	104.2	12.5
**Propiconazole I**	y=1.586x – 32.113	0.998584	102.3	2.94	101.9	5.94
**Trifloxystrobin**	y=1.238x – 86.705	0.998673	114.0	2.41	113.8	5.01
**Propiconazole II**	y=1.983x – 53.710	0.998199	103.8	1.48	115.2	3.20
**p, p' - DDT**	y=2.137x – 148.88	0.998965	102.3	9.72	109.4	8.48
**Quinoxifen**	y=2.559x – 9.855	0.998119	94.8	10.82	93.6	2.55
**Tebuconazole**	y=1.780x – 46.884	0.999526	96.8	10.64	97.8	2.03
**Spiromesifen**	y=0.140x – 24.870	0.992153	94.1	1.85	99.1	1.29
**Endrin ketone**	y=0.216x + 1.631	0.997496	96.6	2.31	96.9	1.35
**Phosmet**	y=3.378x – 136.602	0.992964	96.8	2.65	103.5	1.33
**Bifenthrin**	y=4.292x – 13.071	0.999449	97.3	2.48	101.5	4.94
**Fenoxycarb**	y=1.063x – 31.298	0.990421	98.2	5.14	97.0	1.65
**Metoxychlor**	y=4.591x – 45.894	0.996743	104.5	2.51	105.7	1.19
**Etoxazole**	y=1.010x – 26.167	0.999091	106.8	3.88	108.0	1.83
**Permethrin I**	y=0.549x + 2.312	0.999783	109.4	3.51	105.2	13.51
**Pyridaben**	y=4.694x – 62.409	0.990974	100.9	1.24	103.8	11.08
**Permethrin II**	y=3.477x – 36.719	0.999452	108.7	1.86	97.8	5.18
**Cyflutrin I**	y=0.362x – 0.587	0.999118	100.1	2.13	95.6	1.22
**Cyfluthrin II**	y=0.297x + 1.978	0.994794	105.2	6.80	95.4	1.64
**Cyfluthrin III**	y=0.154x + 18.104	0.992889	104.2	9.16	99.2	1.08
**Cyfluthrin IV**	y=0.361x – 3.247	0.995577	103.2	8.55	92.4	7.16

**Cypermethrin I**	y=0.321x – 11.233	0.997155	94.5	8.52	93.7	7.15
**Cypermethrin II**	y=0.412x – 2.367	0.990974	94.2	1.77	93.0	2.33
**Cypermethrin III**	y=0.294x – 2.384	0.999587	96.2	5.93	95.1	4.73
**Cypermethrin IV**	y=0.389x – 9.559	0.999225	94.1	5.11	96.5	1.86
**Boscalid**	y=3.190x – 10.489	0.997779	94.8	10.49	98.8	2.44
**Ethofenprox**	y=4.831x – 10.478	0.999101	96.9	10.34	102.8	1.97
**Azoxystrobin**	y=0.733x + 17.324	0.996657	97.8	7.19	102.2	3.61

[a] - limit of quantification 0.005 mg/kg. Measurement uncertainty: laboratory value 35%, adopted 50%.

[b] - Recovery (Rec) and precision (RSDr) asssays: spiked blank sediment, n=6

Table S4: Concentrations of elements in the muscle tissue of the black bullhead are expressed in mg kg^{-1} wet weight

	**Pb**	**Cd**	**As**	**Hg**	**Cu**	**Fe**	**Zn**
**Control**	0.14	0.01	nd	nd	0.42	7.64	28.60
**June**	0.08	0.01	nd	nd	0.96	9.72	41.10
**July**	0.08	0.02	nd	nd	0.40	6.83	25.30
**August**	0.13	0.01	nd	nd	0.42	6.53	20.70
**September**	0.12	0.01	nd	nd	0.39	6.31	25.60

*nd- non detected
